# Comparison of oscillometric, non-invasive and invasive arterial pressure monitoring in patients undergoing laparoscopic bariatric surgery – a secondary analysis of a prospective observational study

**DOI:** 10.1186/s12871-022-01619-3

**Published:** 2022-03-28

**Authors:** Jonathan Hansen, Markus Pohlmann, Jan H. Beckmann, Phil Klose, Matthias Gruenewald, Jochen Renner, Ulf Lorenzen, Gunnar Elke

**Affiliations:** 1 Department of Anesthesiology and Intensive Care Medicine, University Medical Center Schleswig-Holstein, Campus Kiel, 24105 Kiel, Germany; 2 Department of General and Abdominal Surgery, University Medical Center Schleswig-Holstein, Campus Kiel, 24105 Kiel, Germany; 3 Department of Anesthesiology and Intensive Care Medicine, Municipal Hospital Kiel, 24116 Kiel, Germany

**Keywords:** Blood pressure, Finger-cuff, Non-invasive monitoring, Nexfin, Clear sight, Obesity, Bariatric surgery, Vascular unloading technique

## Abstract

**Background:**

Oscillometric, non-invasive blood pressure measurement (NIBP) is the first choice of blood pressure monitoring in the majority of low and moderate risk surgeries. In patients with morbid obesity, however, it is subject to several limitations. The aim was to compare arterial pressure monitoring by NIBP and a non-invasive finger-cuff technology (Nexfin®) with the gold-standard invasive arterial pressure (IAP).

**Methods:**

In this secondary analysis of a prospective observational, single centre cohort study, systolic (SAP), diastolic (DAP) and mean arterial pressure (MAP) were measured at 16 defined perioperative time points including posture changes, fluid bolus administration and pneumoperitoneum (PP) in patients undergoing laparoscopic bariatric surgery. Absolute arterial pressures by NIBP, Nexfin® and IAP were compared using correlation and Bland Altman analyses. Interchangeability was defined by a mean difference ≤ 5 mmHg (SD ≤8 mmHg). Percentage error (PE) was calculated as an additional statistical estimate. For hemodynamic trending, concordance rates were analysed according to the Critchley criterion.

**Results:**

Sixty patients (mean body mass index of 49.2 kg/m^2^) were enrolled and data from 56 finally analysed. Pooled blood pressure values of all time points showed a significant positive correlation for both NIPB and Nexfin® versus IAP. Pooled PE for NIBP versus IAP was 37% (SAP), 35% (DAP) and 30% (MAP), for Nexfin versus IAP 23% (SAP), 26% (DAP) and 22% (MAP). Correlation of MAP was best and PE lowest before induction of anesthesia for NIBP versus IAP (*r* = 0.72; PE 24%) and after intraoperative fluid bolus administration for Nexfin® versus IAP (*r* = 0.88; PE: 17.2%). Concordance of MAP trending was 90% (SAP 85%, DAP 89%) for NIBP and 91% (SAP 90%, DAP 86%) for Nexfin®. MAP trending was best during intraoperative ATP positioning for NIBP (97%) and at induction of anesthesia for Nexfin® (97%).

**Conclusion:**

As compared with IAP, interchangeability of absolute pressure values could neither be shown for NIBP nor Nexfin®, however, NIBP showed poorer overall correlation and precision. Overall trending ability was generally high with Nexfin® surpassing NIBP. Nexfin® may likely render individualized decision-making in the management of different hemodynamic stresses during laparoscopic bariatric surgery, particularly where NIBP cannot be reliably established.

**Trial registration:**

The non-interventional, observational study was registered retrospectively at (NCT03184285) on June 12, 2017.

## Background

The standard of care in most low to medium risk surgeries is the oscillometric, non-invasive, intermittent blood pressure measurement (NIBP), which is associated with a lower rate of perioperative complications compared with invasive arterial pressure (IAP) measurements [1, 2]. However, in (morbidly) obese patients, it is impeded by anatomical and physiognomic factors, such as a conical shaped upper arm and increased circumference significantly reducing precision and reliability of NIBP [3, 4]. In cases where a mismatch of cuff size and upper arm circumference hinders NIBP completely, lower arm or lower leg NIBP measurement at the level of the ankle may also reduce the accuracy and seems to be no reliable alternative [5, 6].

Obese and morbidly obese (body mass index, BMI > 40 kg/m^2^) patients are more likely to have a history of type 2 diabetes, arterial hypertension or obstructive sleep apnea syndrome [7–9]. With a steady increase in the prevalence of obesity and bariatric surgery as its most promising treatment [10, 11], precise, easy to use and non-invasive beat-to-beat hemodynamic monitoring is desirable in the perioperative management of these patients. The Nexfin® system is a finger-cuff device combining the vascular-unloading technique with the principle of physiological calibration in order to reconstruct the brachial arterial pressure waveform [12].

Previous studies in different patient populations and clinical settings showed promising results, indicating that Nexfin® is comparable to NIBP [13–18], with the advantage of providing continuous monitoring, yet not proven to be interchangeable with the invasive gold standard [19–22]. In terms of bariatric surgery, studies testing the performance of non-invasive finger-cuff devices are emerging but still infrequent and with divergent results on the agreement with the invasive gold standard [20, 23–27]. Among these studies, only Schuman and coworkers [26] however have analyzed the performance under hemodynamic stresses induced by the on−/off-set of pneumoperitoneum in two different patient postures and were able to show a better agreement for mean and diastolic arterial pressure between finger-cuff and IAP than NIBP and IAP .

Thus, the aim of the present study was to investigate the agreement of arterial pressure measurements between NIBP, Nexfin® and the invasive gold standard using an extended structured protocol mimicking different hemodynamic stresses in the pre-, intra- and postoperative phase of laparoscopic bariatric surgery.

## Methods

### Study design and participants

This is a planned secondary analysis of a single-center prospective observational cohort study where the comparison of continuous cardiac index measurements by Nexfin® and a semi-invasive reference method was previously published [28]. The single-center prospective observational cohort study was conducted at the Department of Anaesthesiology and Intensive Care Medicine and General Surgery, University Medical Center, Schleswig-Holstein, Campus Kiel, the protocol was approved by the local ethics committee of the Christian-Albrechts-University Kiel (file number: A 132/14). Written informed consent was obtained in advance from all participants. Inclusion criteria were adult patients with an indication for elective laparoscopic bariatric surgery, a BMI ≥ 30 kg/m^2^, an ASA (American Society of Aaesthesiologists) class ≥ II and written informed consent for study participation. Since not only morbidly obese patients defined as a BMI ≥ 40.0 kg/m^2^ or 35.0 and 39.9 kg/m^2^ with comorbidities, but also obese patients with BMI > 30 and < 35 kg/m^2^ and type 2 diabetes may have been scheduled for bariatric surgery [7], the BMI ≥ 30 kg/m^2^ criterion was selected. Pre-existing cardiac arrhythmias, peripheral arterial vascular disease Fontaine stadium > 2, pre-existing aortic aneurysm > 4,5 cm and cognitive or linguistic barriers were defined as exclusion criteria.

### Arterial pressure measurements and anesthesia

A description of the study participants’ instrumentation, hemodynamic monitoring and anesthesia was already described previously [28].

NIBP measurement using a forearm cuff on both arms was used for all study participants in order to detect physiological as well as pathological blood pressure differences. Non-invasive blood pressure monitoring (DURA-CUF™ GE, Boston, MA, USA) was conducted on the right arm along with relaxometry and pulse oxymetrically measured oxygen saturation. The appropriately sized finger-cuff of the Nexfin® system (BMEYE, Amsterdam, The Netherlands, now licensed as Clearsight® system by Edwards Lifesciences, Irvine, CA, USA) [12] was placed at the middle phalanx of the index finger and the arterial catheter (Arrow R Intl., Reading, PA, USA; Transducer: DPT-6000, CODAN pvb Critical Care GmbH, Forstinning, Germany) in the radial artery under local anesthesia of the left arm. For the IAP transducer, the zero reference point was selected at the patient’s heart height and the height was corrected accordingly to table position changes during the procedure. Initially, a zero measurement against atmospheric pressure was performed to obtain correct blood pressure values and attention was paid to an undamped pulse pressure curve. The Nexfin® system was connected to the wrist unit as well as the heart reference system. This system adjusts the blood pressure to hydrostatic differences between the sensor and the heart level. The instruments were hold next to each other at the same level to adjust them to zero and the heart reference system detectors were placed at finger and heart level. Finally, biometric patient data were entered as applicable in the Nexfin® monitor.

### Definition of measurement time points and data collection

In the pre-, intra- and postoperative phase, the arterial pressure was measured at 16 predefined measurement time points at which hemodynamic changes were likely expected.

The first measurement was performed in the awake, spontaneously breathing patient in neutral position (baseline measurement time point 1). Directly before the induction of general anesthesia, the patients were placed in 30° anti-Trendelenburg positioning (ATP) as per clinical standard for oxygenation improvement and aspiration prophylaxis (measurement time point 2). The next measurements were taken under general anesthesia, but still in ATP (measurement time point 3), and re-positioned to baseline (measurement time point 4). The following three measurement pairs (time points 5, 6 and 7) were taken during 30° passive leg raising (PLR), again in neutral position and after administration of a 500 ml balanced crystalloid solution bolus infusion (Sterofundin® ISO, Braun, Melsungen, Germany). After this preoperative period the patients were transferred to the operating room, where the measurement devices were reconnected and a new zero balance was performed (baseline II, measurement time point 8). Further measurements were taken intraoperatively after the pneumoperitoneum (PP) had been applied to 15 mbar (baseline PP, measurement time point 9), during intraoperative ATP positioning (measurement time point 10) and in that position after another fluid bolus of 500 ml crystalloid solution (measurement time point 11). The last three intraoperative measurements (measurement time points 12–14) were carried out after termination of PP in neutral position, and again in ATP by the end of general anesthesia, followed by a last measurement in neutral position. Measurement time points 15 and 16 were carried out in the post-anesthesia care unit (PACU) upon arrival and after 2 h in ATP posture, before the patient was discharged from PACU. Demographic data including gender, age, height and weight, ASA classification, comorbidities, and type of surgery and anesthesia were collected from all study participants.

### Statistical analysis

The primary objective of the study was to test interchangeability of the test (NIPB or Nexfin®) against the invasive reference method (IAP). As there is no global definition of interchangeability, we chose two commonly accepted criteria to allow approximation: 1) the recommended Association for the Advancement of Medical Instrumentation (AAMI) criterion of a mean difference ≤ 5 mmHg and associated SD of ≤8 mmHg [29], 2) the percentage error (PE), calculated as 1.96SD of bias/[invasive arterial pressure/2] quantifying the relative differences between NIPB or Nexfin vs. IAP as an additional statistical estimate with acceptable cut-off values at 14.7% for SAP, 17.5% for DAP and 18.7% for MAP [30]. According to previous study protocols [19, 28, 31], sample size was determined with *N* = 60 study participants, followed by an intermediate evaluation. For a Bland-Altman analysis, the width w of the confidence interval for the limits of agreement (LOA) was calculated as w = 6.79•σ•1/√n, where n is number of cases and σ is the standard deviation. For a case count of *N* = 60, the result is w = 0.88 • σ and thus considered a sufficiently large number. Normal distribution of the outcomes was checked and verified by visual inspection of the histogram analysis. First, a Spearman correlation analysis of measurement pairs for SAP, DAP and MAP between the monitoring devices was performed. Furthermore, Bland-Altman analysis was used for the comparison of the pressure variables with calculation of the mean difference (bias) and limits of agreement (LOA) defined as the standard deviation (SD) of the bias ±1.96 accounting for repeated measurements [32, 33]. The concordance was calculated as the percentage of measurement pairs with the same direction of change after exclusion of pairs with a change < 5% and calculated as the proportion (percentage) of concordant data pairs to all data pairs with an acceptable ability to show hemodynamic trends when the level of concordance was > 92% [34]. We further sought to evaluate whether precision of measurements would be different under hypo- and hypertensive episodes. Therefore, data pairs of NIBP/IAP and Nexfin®/IAP were divided into two groups of hypotension defined IAP MAP values ≤50 mmHg and hypertension defined as IAP MAP values ≥70 mmHg.

A *P* value of < 0.05 was considered as statistically significant. Statistical analysis was performed using GraphPad Prism 5, GraphPad Software Inc., San Diego, CA, USA.

## Results

**Fig. 1:**
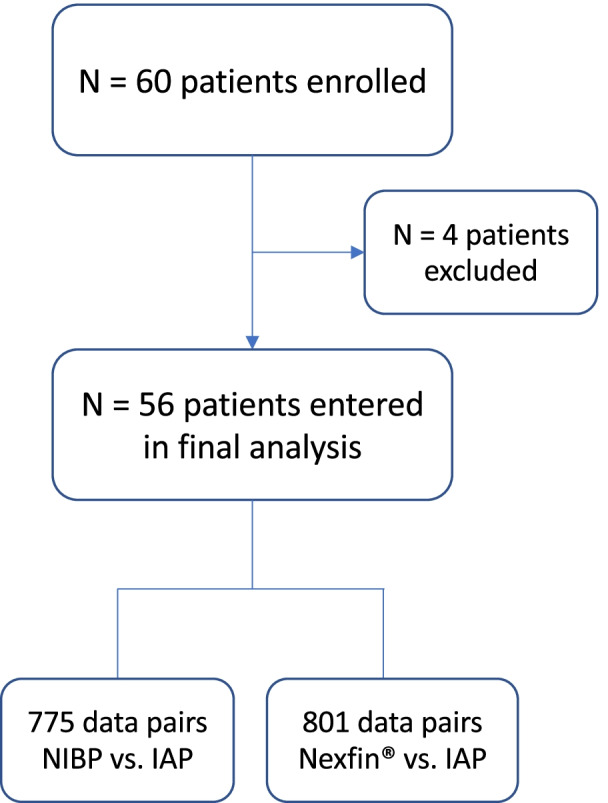
Study participant flow diagram

Sixty patients were enrolled in the study with four patients being excluded from the statistical analysis due to insufficient data acquisition (Fig. 1). Table 1 summarizes baseline characteristics of the study participants. 86.6% (*N* = 832) of all planned measurements could be realized. Reasons for lack of measurement data pairs (*N* = 128) were mostly attributed to intraoperative events preventing measurements (*N* = 90) and challenges in function and handling of the measurement device, of which the NIBP device failed significantly more frequently (*N* = 31) than the IAP (*N* = 2) or Nexfin® monitoring (*N* = 5). All measurements were pooled and included in the statistical analysis.

**Table 1:** Study participants characteristics, type of bariatric surgery and anesthesia

**Number of study participants**	**60**
**Age, years**	46.5 (12.1)
**Gender, N (%)**	female 44 (73) male 16 (27)
**Height, cm**	172 (10)
**Body weight, kg**	147 (27)
**Body mass index, kg/m**^**2**^	49.2 (5.7)
**Comorbidities**	
Arterial hypertension, N (%)	37 (61)
Diabetes mellitus, N (%)	23 (38)
**Type of bariatric surgery**	
Gastric bypass, N	31
Sleeve gastrectomy, N	24
Gastric banding explantation, N	1
Single anastomosis duodeno-ileal bypass-sleeve gastrectomy (SADI-S), N	4
**Type of general anaesthesia**	
Total intravenous anaesthesia with propofol and remifentanile	19
Balanced anaesthesia with sevoflurane and remifentanile/desflurane and remifentanile	31/10

Variables are expressed as mean (SD) unless otherwise indicated in the table

### NIBP and Nexfin® versus IAP measurement

**Fig. 2:**
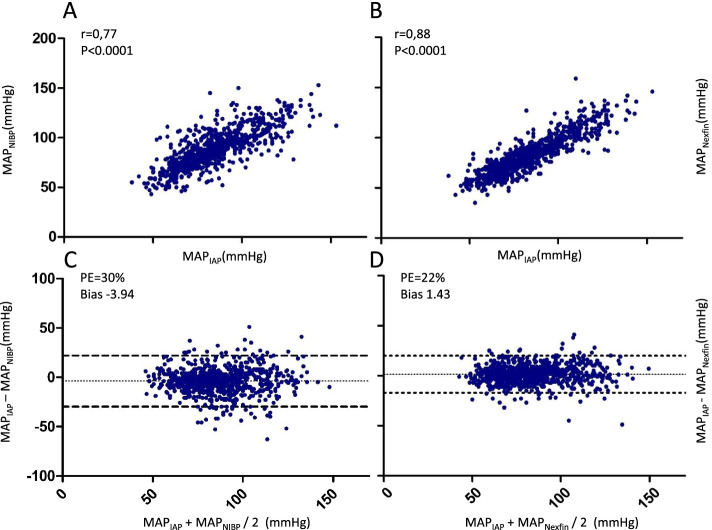
Pooled correlation and Bland-Altman analyses for all MAP data pairs measured by NIBP and Nexfin® versus IAP. Panels **A** and **B**: NIBP (**A**) or Nexfin® (**B**) (y-axis) derived values are plotted against IAP values (x-axis), with correlation coefficient (r) and *P* value displayed in the diagrams. Panels **C** and **D**: Bland-Altman plots, where differences of IAP and NIBP (**C**) or Nexfin® (**D**) (y-axis) are plotted against their common mean (x-axis). The bold dotted lines display the limits of agreement, where 95% (bias ±1.96 standard deviation of the difference) of all measurements are located. The middle line shows the mean difference (bias). Percentage error and bias are displayed in the diagrams. MAP: Mean arterial pressure, PE: Percentage error, IAP: Invasive arterial pressure, NIBP: Non-invasive oscillometric blood pressure, r: Correlation coefficient

Table 2 summarizes the pooled correlation and Bland-Altman analyses for MAP measured by NIBP and Nexfin® versus IAP, respectively according to each measurement time point. Figure 2 shows Bland-Altman and correlation analyses for the respective pooled results for MAP over all measurement time points.

**Table 2:** Summary of correlation and Bland-Altman analyses of MAP values measured by NIBP or Nexfin® compared with IAP according to each perioperative measurement time point

Time point	NIBP vs. IAP					Nexfin® vs. IAP				
	Data pairs N	Correlation coeffcient	Bias (SD) mmHg	LOA mmHg	PE %	Data pairs N	Correlation coeffcient	Bias (SD) mmHg	LOA mmHg	PE %
**1**	52	0.56*	-2.5 (14.6)	-31.2 to 26.2	27.9	52	0.74*	4.3 (10.4)	-16.2 to 24.9	20.7
**2**	52	0.72*	-5.5 (12.5)	-29.8 to 20.0	24.1	53	0.79*	1.6 (10.9)	-19.7 to 23.0	21.9
**3**	52	0.63*	-10 (12.4)	-34.4 to 14.5	33.6	54	0.76*	-1.7 (9.6)	-20.5 to 17.0	27.3
**4**	50	0.59*	-5.9 (13.9)	-33.3 to 21.4	34	54	0.82*	4.2 (8.8)	-13.2 to 21.6	23.1
**5**	51	0.48	-2.2 (14.9)	-31.5 to 26.7	36.8	53	0.74*	6.8 (8.9)	-10.6 to 24.2	23.3
**6**	27	0.62	-3.6 (14.2)	-31.7 to 24.4	39.3	28	0.83*	2.2 (6.4)	-10.4 to 14.8	18.5
**7**	53	0.71*	-3.3 (11.3)	-25.2 to 18.8	29.4	55	0.88*	5.1 (5.9)	-6.4 to 16.7	17.2
**8**	54	0.65*	-5.7 (11.2)	-28.0 to 16.4	31.9	55	0.85*	0.8 (6.9)	12.6 to 14.4	20.4
**9**	54	0.76*	-2.4 (12.7)	-27.2 to 22.2	30.9	56	0.90*	1.6 (7.9)	-13.8 to 17.1	19.7
**10**	53	0.76*	-4.6 (11.5)	-27.3 to 18.1	30.2	54	0.84*	-3.3 (8.7)	-20.5 to 13.7	23.0
**11**	55	0.68*	-7.1 (12.7)	-32.2 to 17.9	30.0	55	0.83*	-1.0 (8.3)	-17.4 to 15.2	20.2
**12**	55	0.70*	-8.0 (10.0)	-27.8 to 11.9	25.5	56	0.79*	-3.6 (7.4)	-18.2 to 10.8	19.4
**13**	52	0.70*	-1.4 (11.7)	-24.2 to 21.4	26.0	55	0.89*	3.3 (6.4)	-9.0 to 15.7	14.6
**14**	47	0.70*	-4.9 (15.6)	-35.4 to 25.5	29.7	51	0.78*	2.7 (13.1)	-22.9 to 28.3	25.6
**15/16**	72	0.59*	5.1 (11.5)	-17.7 to 27.9	21.8	70	0.62*	-0.3 (11.4)	-22.6 to 22.0	20.7

*P < 0.05

The pooled correlation coefficient between NIBP and IAP for MAP was 0.77 (SAP 0.63, DAP 0.72). Bland-Altman plots corroborated a correlation with a PE of 30% and a bias of − 3.94 mmHg for MAP (SAP: PE 37%, bias − 3,5 mmHg, DAP: PE 35%, bias − 4 mmHg). Pooled correlation between Nexfin® and IAP over all measurement time points was 0.88 for MAP (SAP: 0.87, DAP: 0.80). Bland Altman analysis showed a PE of 22% and a bias of 1.43 mmHg for MAP (SAP PE: 23%, bias 12 mmHg, DAP PE 26%, bias 0.7 mmHg).

**Fig. 3:**
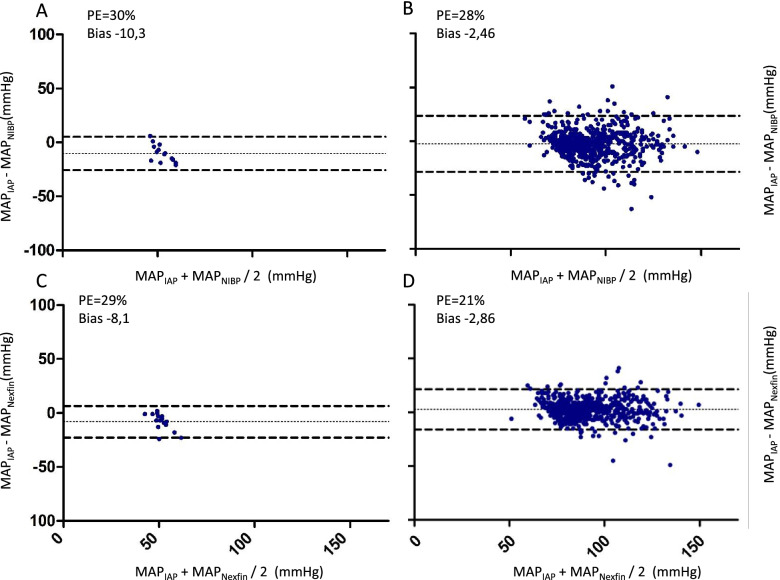
Bland Altman analyses for hypotension and hypertension. Bland-Altman Plots of all values, where MAP recorded by IAP was ≤50 mmHg (**A** and **C**) or > 70 mmHg (**B** and **D**). Differences of IAP and Nexfin®/NIBP (y-axis) are plotted against their common mean (x-axis). Bold dotted lines display the limits of agreement, where 95% (bias±1.96 standard deviation of the difference) of all measurements are located. The middle line shows the mean difference (bias). Percentage error and bias are displayed in the diagrams. MAP: Mean arterial Pressure, PE: Percentage error, IAP: Invasive arterial pressure

With respect to hypotension, PE was comparable between NIBP (30%) and Nexfin® (29%) vs. IAP whereas during hypertension, PE was lower for NIBP (28%) and Nexfin® (21%) vs. IAP (Fig. 3).

### Trending

**Fig. 4:**
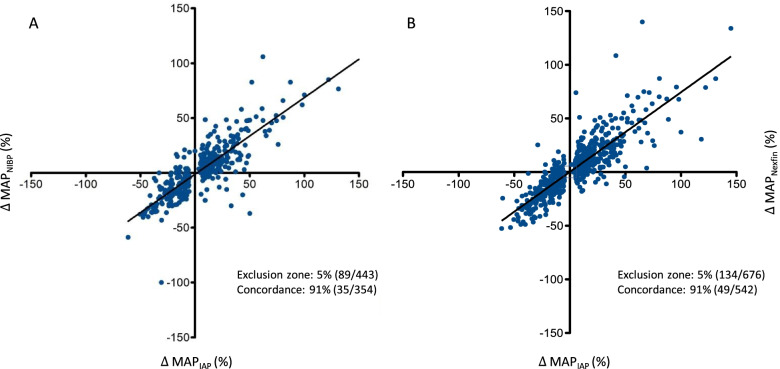
Trending analysis for Nexfin® or NIBP measured MAP. Four square plots of the concordance for MAP values recorded by NIBP (Panel **A**) or Nexfin® (Panel **B**) vs. IAP. The y-axis shows changes (as percentages) of IAP, the x-axis shows changes (as percentages) of Nexfin® or NIBP derived arterial pressure measurements for the total data sample. The left lower and right upper quadrants include all arterial pressure values with the same (negative or positive) change. Changes with less than 5% were excluded from portrayal and statistical analysis. One data pair lies outside the depicted range in Panel **A**. MAP: Mean arterial pressure, NIBP: Non-invasive oscillometric blood pressure, IAP: Invasive arterial pressure

Table 3 compares trending capability for SAP, DAP and MAP measured by NIBP and Nexfin® for two subsequent measurement time points mimicking changes in hemodynamics. Figure 4 presents an overview of trending ability for NIBP or Nexfin® measured MAP over all measurement time points. Regarding NIBP, MAP values showed highest trending capability, reaching a concordance rate of > 92% at 3 measurement time points (SAP and DAP at 2 time points, respectively) and a concordance rate of 90% throughout all time points (SAP 85%, DAP 89%). MAP trending measured by Nexfin® revealed a concordance rate of > 92% at 5 subsequent measurement time points (SAP at 3, DAP at 2 time points) and an overall concordance rate of 91% (SAP 90%, DAP 86%).

**Table 3:** Concordance rates for every measurement time point compared to its preceding measurement time point

Time points	SAP (NIBP)	DAP (NIBP)	MAP (NIBP)	SAP (Nexfin®)	DAP (Nexfin®)	MAP (Nexfin®)
**1→2**	27/52 68% (8/25)	20/51 68% (16/31)	28/53 48% (13/25)	27/52 40% (15/25)	20/52 33% (21/32)	28/53 32% (17/25)
**2→3**	1/49 93% (3/48)	5/49 98% (1/44)	3/52 87% (6/49)	1/52 97% (1/51)	5/52 100% (0/47)	3/52 97% (1/49)
**3→4**	13/48 82% (6/35)	13/48 89% (4/35)	10/48 89 % (4/38)	13/53 88% (5/40)	13/53 85% (6/40)	11/53 88 % (5/42)
**4→5**	21/50 68% (9/29)	16/50 75% (8/34)	18/50 84% (5/32)	22/53 89% (3/31)	18/53 86% (5/35)	20/53 78% (7/33)
**5→6**	7/27 90% (2/20)	9/27 82% (3/18)	8/27 84% (3/19)	7/28 89% (2/21)	9/28 95% (1/19)	8/27 94% (1/19)
**6→7**	10/26 87% (2/16)	5/26 89% (2/21)	6/25 94% (1/19)	11/27 94% (1/16)	5/27 82% (4/22)	6/26 90% (2/20)
**7→8**	7/52 84% (7/45)	8/52 91% (4/44)	5/51 91% (4/46)	8/54 87% (6/46)	8/54 88% (5/46)	6/54 95% (2/48)
**8→9**	11/55 84% (7/44)	10/56 91% (3/46)	9/53 88% (5/44	12/55 91% (4/43)	10/55 90% (4/45)	10/55 93% (3/45)
**9→10**	11/51 95% (2/40)	15/52 92% (3/37)	11/51 97% (1/40)	11/54 85% (6/43)	15/54 77% (9/39)	11/55 90% (4/44)
**10→11**	11/54 84% (7/43)	10/54 83 (7/44)	11/53 90% (4/42)	11/55 89% (5/44)	11/55 83% (7/44)	11/55 90% (4/44)
**11→12**	8/53 87% (6/45)	9/53 85% (6/44)	7/51 93% (3/44)	8/55 90% (4/47)	9/55 90% (4/46)	7/55 93% (3/48)
**12→13**	3/52 90% (5/49)	3/52 80% (10/49)	4/51 91% (4/47)	3/55 93% (3/52)	3/55 91% (4/52)	4/55 96% (2/51)
**13→14**	7/49 86% (6/42)	11/50 90% (4/39)	11/48 91% (3/37	9/51 93% (3/42)	11/51 88% (5/40)	11/51 92% (3/40)
**15→16**	9/36 91% (3/33)	16/36 60% (8/20)	13/36 91% (2/23)	9/35 62% (10/26)	16/35 62% (7/19)	13/34 71% (6/21)

Measurements with a measured delta of less than 5% to the reference IAP were excluded (upper left number), followed by the total number of all feasible measurements during the specific time point. Changes with a unidirectional (negative or positive) delta are displayed as percentages (lower left number) followed by the number of measurements with contradicting delta out of all data pairs with a delta of more than 5%. Example: After the first hemodynamic stress (30° Anti-Trendelenburg positioning), 27 measurements of systolic arterial pressure measured by NIBP varied less than 5% compared to the preceding measurement out of a total of 52 feasible measurements. Out of the remaining 25 significant (eg. greater than 5% delta) measurements, 8 showed contradictional changes of the invasive arterial pressure, indicating reliability of the tested measurement method in 17 cases, resulting in a concordance rate of 68%

## Discussion

In this secondary analysis of a prospective observational cohort study including 56 patients with a mean BMI of 49.2 kg/m^2^ undergoing laparoscopic bariatric surgery, arterial pressure measurements by NIBP and Nexfin® non-invasive finger-cuff system were compared to the invasive arterial gold-standard at 16 predefined perioperative time points. Overall, interchangeability for absolute arterial pressure values in terms of the AAMI criterion and PE could neither be shown for NIPB nor Nexfin®. However, Nexfin® systematically surpassed NIBP performance over all measurement time points. Precision of NIBP and Nexfin® measurement increased under adequate intravascular blood volume distribution, cardiac preload and hence improved hemodynamic conditions mimicked by fluid bolus administration and posture changes in this study. Regarding trending ability, Nexfin® showed an overall concordance rate of 86–91%, indicating a clinically acceptable performance under hemodynamic changes yet only slightly better than NIBP.

Concerning interchangeability between non-invasive finger-cuff devices and the invasive gold-standard, a recent meta-analysis of 24 studies including 1164 surgical or critically ill patients showed a tendency to oppose equivalence in terms of PE while the overall pooled results indicated that both techniques are not interchangeable in the studied heterogenous patient population with a high heterogeneity in measurement performance and statistical analyses [19].

With regard to morbidly obese patients undergoing bariatric surgery, four observational studies have been previously published focussing on the performance of the Nexfin® (Clearsight®) finger-cuff device for arterial pressure monitoring [20, 24, 26, 27]. Pouwels et al. were unable to demonstrate interchangeability between the Nexfin® system and NIBP measurements in 33 patients with a mean BMI of 42 kg/m^2^, yet both measurements were not validated against IAP measurement [24]. A further study by Rogge and coworkers using the Clearsight® system in comparison to IAP in 35 patients with a median BMI of 47 kg/m^2^ found that the accuracy and precision of the vascular unloading technique was good for MAP (bias 1.1 mmHg LOA: − 13.5 to 15.6 mmHg) and DAP (bias 0.8 mmHg, LOA − 12.9 to 14.4 mmHg), but only moderate for SAP (bias 6.8 mmHg, LOA − 14.4 to 27.9 mmHg), missing the predefined AAMI criterion of interchangeability. Trending capability was good with concordance rates of 88–93%. In contrast to our study, measurements were recorded continuously using an interface cable and recording software for 45 min independently of intraoperative events and cleared of obvious artifacts subsequently by visual inspection [20]. The prospective study by Eley et al. in 30 participants with a median BMI of 50.2 kg/m^2^ was unable to show interchangeability of intraoperatively measured absolute arterial pressure values with an observed bias (SD) of 14.3 mmHg (14.1) for SAP, 2.6 mmHg (10.8) for DAP and 5.2 mmHg (10.9) for MAP as compared to IAP [27]. The recent study by Schumann and coworkers revealed an overall good agreement between Nexfin® and IAP monitoring in 90 patients (mean BMI 47 kg/m^2^) with bariatric surgery. In their study, measurements were obtained within 15 min before start of PP in the horizontal position, 3, 15, 30, and 45 min after the beginning of PP in the 30° reverse Trendelenburg position and 3 min after end of PP in horizontal posture. Furthermore, NIBP and Nexfin® were recorded at the ipsilateral arm as opposed to the contralateral measurement in our study. DAP showed best precision (mean difference 0 ± 11 mmHg), followed by MAP (mean difference − 1 ± 11 mmHg) and SAP (mean difference − 7 ± 14 mmHg) between finger-cuff and IAP measurements while NIBP measurement was significantly less precise [26]. Concordance between changes in finger-cuff and IAP measurements was high with 88% for MAP, which is in line with our findings where concordance was even higher with 86–91% over all time points and with trending above the Critchley criterion for delta values for single subsequent measurement time points. Perioperative arterial pressure values were pooled in the above studies, and except for the study by Schumann et al. no tests were performed under different hemodynamic stresses or perioperative phases, respectively. Two further studies have compared the CNAP® system, which measures arterial pressure beat-to-beat by the volume clamp technology, with IAP measurements [23, 25] in patients undergoing bariatric surgery. Tobias and coworkers investigating 18 severely obese adolescents (BMI range from 37.9 to 74.7 kg/m^2^) and young adults during robotic, laparoscopic-assisted bariatric surgery and were able to only show a clinically useful trend ability of the arterial pressure values for CNAP while accuracy for absolute values failed the AAMI criterion [25]. The other prospective observational study by Rogge and coworkers including 29 patients (mean BMI 48.1 kg/m^2^) undergoing laparoscopic bariatric surgery correspondingly found good trending ability for the CNAP® system compared to continuous IAP measurements obtained with a radial arterial catheter, but again no interchangeability for absolute values [23].

In essence, while results from our and the 5 published studies on interchangeability for absolute values remain inconclusive with a notion that Nexfin is slightly more precise, overall trend behavior of the finger-cuff technology was consistently shown to be clinically useful. The latter was also shown for various subsequent measurement time points in our study where hemodynamic changes become evident in the perioperative phases of laparoscopic bariatric surgery, providing useful clinical information on the presence of arterial pressure variation and its management. Finally, we further analyzed precision in the range of hypo- and hypertension as this is clinically even more relevant than information on “global performance”. With focus on hypotension, Nexfin® and NIBP tended to overestimate arterial pressure, possibly leading to false security in clinical practice while during hypertensive episodes no difference was observed. However, data pairs for hypotension in our study were too limited to be conclusive, most likely because a direct therapeutic intervention was performed for patient safety. In a previous study in 30 awake carotid endarterectomy patients, Noto and coworkers showed that precision of Clearsight® measurements were not interchangeable below an average MAP cut-off value of 69 mmHg [22].

A limitation of our study is that the sample size of 60 patients deviates from the recently updated standard for the validation of blood pressure measuring devices by the AAMI, the European Society of Hypertension (ESH) and the International Organization for Standardization (ISO) collaboration where where a minimum sample size of 85 participants is recommended for studies validating non-invasive sphygmomanometers [29]. However, there are still no specific recommendations available on how to evaluate continuous, non-invasive blood pressure monitoring techniques, including methodological criteria of an adequate effect and sample size which is also acknowledged in the recent AAMI/ESH/ISO collaboration statement. As intended, our cohort reflects a typical morbidly obese patient population with an indication for laparoscopic bariatric surgery, being relatively young, rather female with most of them having a history of arterial hypertension or diabetes. Thus, another limitation of our study is the generalizability particularly to older obese patients with (advanced) peripheral artery disease or atrial fibrillation. The effect of atrial fibrillation on Nexfin® measurements is not fully understood, although Nexfin® showed reliable measurements in one recent study [35]. Another limitation is the arrangement of devices on each patient: NIBP measurement was installed on the right patient upper arm, IAP and Nexfin were mounted on the left patient arm. Intraoperative patient positioning could have caused artefacts, leading to a false high difference between the IAP and NIBP measurements. The indwelling radial catheter could also have changed the distal bloodstream, impacting the plethysmography-based finger-cuff measurement algorithm. The fact that NIBP measures the brachial BP but Nexfin measures the digital artery BP and reconstructs brachial BP might lead to a systematic error, increasing a potential gap between obtained values. Lastly, we did not measure the range of mid-arm circumference or systematically adjusted for the range of NIBP cuff sizes used. Schumann et al. [26] were able to show that NIBP performed better on the forearm than upper arm and lower leg with regard to absolute and trending agreement as compared to IAP. However, Eley and coworkers argued that NIBP cuffs were not based on the mid-arm circumference, as recommended by the American Heart Association [36] and that patient allocation to correctly sized cuffs is essential for a validation [27]. Our study did not focus on a cost-effectiveness analysis for the different blood pressure monitoring methods used in the bariatric population and non-manufacturer derived data on this subject is yet limited [37, 38]. The acquisition costs could be a barrier to the introduction in anesthesia departments [39]. Further studies may focus on whether the higher acquisition costs together with personnel and material costs of the different methods impact patient-centered clinical outcomes.

Strengths of our study include the examination of different hemodynamic stresses typically occurring throughout the perioperative course of laparoscopic surgery, particularly due to different postures and on/off-set of PP. Mimicking those physiological stresses using a structured protocol in the pre-, intra- and postoperative phase enhances the “real-life” comparison of the device performance.

## Conclusion

In the perioperative management of patients undergoing laparoscopic bariatric surgery, our study indicates that NIBP and Nexfin® derived absolute arterial pressure recordings were not interchangeable with IAP, but Nexfin® was more precise than NIBP. However, a good trending ability even under different hemodynamic stresses was found. Thus, Nexfin® may serve clinically useful to detect arterial pressure changes and render perioperative hemodynamic treatment, particularly in those individuals where NIBP cannot be reliably established.

## Data Availability

The datasets used and/or analyzed during the presented study are available in an anonymous fashion from the corresponding author on reasonable request.

## Abbreviations

AAMI: Association for the Advancement of Medical Instrumentation
ASA: American Society of Anesthesiologists
ATP: Anti-Trendelenburg posture
BMI: Body mass index
CI: Cardiac index
DAP: Diastolic arterial pressure
ESH: European Society of Hypertension
HR: Heart rate
IAP: Invasive arterial pressure
ISO: International Organization for Standardization
LOA: Limits of agreement
MAP: Mean arterial pressure
NIBP: Non-invasive (oscillometric) blood pressure
PACU: Post-anesthesia care unit
PE: Percentage error
PLR: Passive leg raising
PP: Pneumoperitoneum
SAP: Systolic arterial pressure
SD: Standard deviation
w: Width
